# Maternal exposure to housing renovation during the periconceptional period and the risk of offspring with isolated congenital heart disease: a case-control study

**DOI:** 10.1186/s12940-023-00990-z

**Published:** 2023-04-18

**Authors:** Pengfei Qu, Doudou Zhao, Mingxin Yan, Danmeng Liu, Ruo Zhang, Shanshan Li, Leilei Pei, Hong Yan, Lingxia Zeng, Shaonong Dang

**Affiliations:** 1 Translational Medicine Center, Northwest Women’s and Children’s Hospital, Xi’an, 710061 China; 2 Department of Epidemiology and Biostatistics, School of Public Health, Xi’an Jiaotong University Health Science Center, Xi’an, 710061 China; 3 Department of Endocrinology, The Second Affiliated Hospital of Xi’an Jiaotong University, Xi’an, 710004 China; 4 School of Public Health, Shandong First Medical University & Shandong Academy of Medical Sciences, Jinan, 250117 China

**Keywords:** Case-control study, Congenital heart disease, Housing renovation, Infant, Pregnant women

## Abstract

**Background:**

Congenital heart disease (CHD) is the most prevalent birth defect in recent decades. The aim of this research was to examine the association between maternal housing renovation exposure during the periconceptional period and isolated congenital heart disease (CHD) in their offspring.

**Methods:**

A multi-hospitals case-control study was conducted from six tertiary A hospitals in Xi’an, Shaanxi, Northwest China based on questionnaires and interviews to address this question. The cases included fetuses or newborns diagnosed with CHD. Controls consisted of healthy newborns without birth defects. In total, 587 cases and 1180 controls were enrolled in this study. The association between maternal periconceptional housing renovation exposure and isolated CHD for offspring was assessed by estimating odds ratios (OR) with multivariate logistic regression models.

**Results:**

After adjusting for potential confounding variables, it was found that maternal exposure to home improvement projects was associated with a higher probability of isolated CHD in offspring (adjusted OR: 1.77, 95% CI: 1.34, 2.33). Additionally, the risk of the ventricular septal defect (VSD) and patent ductus arteriosus (PDA) for CHD types was significantly associated with maternal exposure to housing renovations (VSD: adjusted OR = 1.56, 95% CI: 1.01, 2.41; PDA: adjusted OR = 2.50, 95% CI: 1.41, 4.45).

**Conclusions:**

Our study suggests that maternal exposure to housing renovation during the periconceptional period was associated with an increased risk of isolated CHD in offspring. Consequently, it would be beneficial to avoid living in a renovated home from 12 months before pregnancy through the first trimester to lower isolated CHD in infants.

**Supplementary Information:**

The online version contains supplementary material available at 10.1186/s12940-023-00990-z.

## Introduction

Congenital heart disease (CHD) refers to the malformation caused by abnormal cardiovascular development in the developing fetus. CHD is the most prevalent birth defect, leading to miscarriage, stillbirth, and infant deaths [1]. Worldwide, the incidence of CHD is about 4–10 per 1,000 live births and is increasing [2]. According to China’s birth defect surveillance data, the occurrence of CHD reached 8–10% in live births, and 150,000 newborns are born each year with CHD [3]. However, because the epidemiology studies and data on CHD from low-and-middle-income countries are far more limited, the CHD prevalence is frequently underestimated in these countries [4]. Therefore, CHD has become a significant public health problem worldwide and in China. Although some studies suggested that CHD may be developed during the first trimester of pregnancy due to genetic factors, environmental factors, or gene-environment interaction [5, 6], the pathogenesis of CHD is still unknown [7].

More Chinese have relocated into modern homes after renovation in order to improve their living standards as a result of the social economy’s rapid development and urbanization. Unfortunately, with time, decorative materials emit volatile and organic pollutants that have an adverse effect on human health. Several studies have revealed that maternal exposure to organic solvents (toluene and xylene) potentially increased the risk of abnormal fetal development [8–11]. Additionally, there is evidence in experimental model systems that prenatal exposure to environmental pollutantspolycyclic (trichloroethylene or aromatic hydrocarbons) causes developmental abnormalities in the hearts of avian embryos and embryos in animals [9, 12–14]. However, there is a paucity of data to demonstrate the association between maternal housing renovation exposure during the periconceptional period and isolated CHD risk [15]. In order to investigate the relationship between maternal housing remodeling exposure during the periconceptional period and the risk of isolated CHD in their offspring, a hospital-based case-control research was carried out in Xi’an, China.

## Methods

### Study design and participants

We conducted a case-control study in six tertiary A hospitals from January 2014 to December 2016 in Xi’an, Shaanxi province, China. At the obstetrics departments, neonates and mothers were chosen as cases and controls after childbirth. Inclusion criteria and exclusion criteria for the cases were as follows: (1) Singleton pregnancy; (2) End of pregnancy was between January 2014 and December 2016; (3) Mothers whose fetuses or children were diagnosed with isolated CHDs from 28 weeks of pregnancy to 28 days after birth, and the pregnancy outcomes with live birth, stillbirth or termination after fetal anomaly were included in case group; (4) All children diagnosed with a patent ductus arteriosus (PDA) born at term; (5) The case group excluded any fetus or child with birth defects other than CHD.

Two controls were selected after one case was recruited. Inclusion criteria and exclusion criteria for the controls were as follows: (1) Controls came from the same year as the case; (2) Controls came from the same hospital as the case; (3) In the control group, live births with a singleton pregnancy were included; 4)The control group excluded mothers whose fetus or child with birth defects. In the case group, 603 participants were surveyed with a no-reply rate of 2.65% (16 cases). In the control group, 1216 participants were surveyed with a no reply rate of 2.96% (36 cases). Finally, the analysis included 587 cases and 1180 controls who had completed the questionnaires.

### Data collection

A standardized questionnaire was designed by the Department of Epidemiology and Health Statistics, School of Public Health, Xi’an Jiaotong University. The questionnaire included sociodemographic characteristics, life behaviors, nutrient supplements, family history of birth defects, and environmental factors during the periconceptional period. Mostly through hospital medical records, additional details, such as pregnancy history, disease and medication usage throughout pregnancy, and delivery status, were acquired. Furthermore, all participants were interviewed by face-to-face during their stay in the delivery hospital. And the interviewers were uniformly trained staff from the Department of Epidemiology and Health Statistics, Xi’an Jiaotong University.

We established a team of experts on CHD diagnosis, and the expert team comprised several senior medical technicians from the congenital heart surgery, ultrasound, obstetrics, and gynecology departments of the Northwest Women’s and Children’s Hospital and the First Affiliated Hospital of Xi’an Jiaotong University. The diagnoses of CHD were based on the ICD-10 by the expert team.

### The definitions of housing renovation

This study collected housing renovation exposure during the periconceptional period from self-reporting by the mothers in question. Housing renovation exposure was defined as the usage of laminated board, plywood, ceramic tile, marble, oil-based paint, latex or acrylic coating, wallpapers, or carpets during house or work renovations that occurred from one year before to pregnancy through the first trimester of pregnancy.

### Covariates

Potential confounders correlated with CHD such as demographic characteristics, life behaviors and nutrient supplements were also collected for the participants. These variables included maternal age, maternal body mass index (BMI), education (primary school and below, junior middle school, high middle school, above high middle school), ethnicity (Han, other), residence (rural, urban), family wealth index (low, medium, high), parity (0, 1, ≥ 2), abortion history (yes, no), family history of birth defects (yes, no), active or passive smoke (yes, no), drinking (yes, no), folic acid supplement (yes, no), fever (yes, no), taking medicine (yes, no), gestational diabetes mellitus (GDM) (yes, no), TORCH infections (yes, no), industrial exposure (yes, no).

The early pregnancy period was defined as the first trimester of pregnancy (up to 12 weeks gestation). The 12 months before conception through the first trimester of pregnancy were referred to as the periconceptional period. Active smoke was defined as ≥ 1 cigarette per week for three consecutive months during the periconceptional period. Passive smoke was defined as ≥ 15 min of smoke inhalation per day for one month during early pregnancy. Drinking refers to drinking alcoholic products (beer, red wine, or white wine) at least one time during early pregnancy. The folic acid supplement was defined as the regular use of folic acid (≥ 400 µg folic acid per day) from 3 months before pregnancy through the first trimester of pregnancy for more than one consecutive month. Fever was defined as febrile episodes (> 38 °C) at least once during early pregnancy. Taking medicine was defined as taking any drugs such as antibiotics or hormones during early pregnancy. GDM was defined as fasting plasma glucose (FPG) ≥ 5.1 mmol/L, or one h plasma glucose ≥ 10.0 mmol/L or two h plasma glucose ≥ 8.5 mmol/L in a 75 g oral glucose tolerance test (OGTT) at 24–28 weeks of gestation. TORCH infections were defined as toxoplasmosis, rubella, cytomegalovirus, and herpes simplex infections. Living within a 10-kilometer radius of mines, paper mills, cement plants, power plants, pesticide factories, and fertilizer factories during the periconceptional period was considered to be industrial exposure.

### Statistical analysis

The participants’ baseline characteristics are summarized using counts and proportions for categorical variables. The chi-squared test or Fisher’s exact test was performed to compare categorical variables. The family wealth index was created from a database of household income, assets, and facilities using the principle component analysis approach. This index was categorized into tertials (low, medium, high) that indicated the poorest, middle-class, and wealthiest households. The univariate and multivariate logistic regression models were used to assess the effect of housing renovation on isolated CHD and the main subtype of CHD (ventricular septal defect (VSD), atrial septal defect (ASD), PDA, atrioventricular septal defect (AVSD), and tetralogy of Fallot (TOF)). Model 2 adjusted baseline demographic characteristics (maternal age, BMI, education, ethnicity, residence, family wealth index). Model 3 adjusted all baseline covariates (maternal age, BMI, education, ethnicity, residence, family wealth index, parity, abortion history, family history of birth defects, active or passive smoke, drinking, folic acid supplement, fever, taking medicine, GDM, TORCH infections, and industries exposure).

A sensitivity analysis was conducted using two approaches to assess the robustness of our findings. To examine the potential heterogeneity of a housing renovation effect on isolated CHD, we estimated the effects of housing renovation in subgroups of different maternal characteristics. Additionally, propensity scores matching (1:1) was used for controlling confounding factors, and further assessing the effect of housing renovation on isolated CHD.

All statistical analysis in this study was completed through SAS9.4. A two-tailed P-value below 0.05 was considered statistically significant.

## Results

### Participant characteristics

A total of 587 mothers with isolated CHD offspring (case group) and 1180 mothers with normal offspring (control group) were analyzed in the study. The characteristics of participants were compared between the case group and control group (Table 1). Compared to the control group, the case group was more likely to have to live in rural areas, had higher BMI, parity, family history of birth defects, active or passive smoking, drinking, taking medicine, TORCH indections, and industries exposure during the periconceptional period. Finally, the case group was more likely to have a lower age, education level and family wealth index, less Han ethnicity, and folic acid supplementation.

**Table 1:** Baseline characteristics of participants between case group and control group

	Case group n = 587	Control group n = 1180	t/χ^2^ /Z value	*P* value
Maternal age, n (%)	28.24 ± 5.02	29.00 ± 4.27	-3.790	< 0.001^a^
Maternal BMI	20.73 ± 2.54	20.41 ± 2.04	-2.413	0.016 ^a^
Education, n (%)				
Primary school and below	8 (1.36)	5 (0.42)	171.408	< 0.001
Junior middle school	27 (4.60)	111 (9.41)		
High middle school	227 (38.67)	147 (12.46)		
Above high middle school	325 (55.37)	917 (77.71)		
Ethnicity, n (%)				
Han	572 (97.44)	1172 (99.32)	10.755	0.001
Other	15 (2.56)	8 (0.68)		
Residence, n (%)				
Rural	390 (66.44)	391 (33.14)	176.287	< 0.001
Urban	197 (33.56)	789 (66.86)		
Family wealth index, n (%)				
Low	309 (52.64)	325 (27.54)	117.732	< 0.001
Medium	161 (27.43)	391 (33.14)		
High	117 (19.93)	464 (39.32)		
Parity, n (%)				
0	360 (61.33)	947 (80.25)	83.038	< 0.001
1	200 (34.07)	223 (18.90)		
≥ 2	27 (4.60)	10 (0.85)		
Abortion history, n (%)				
Yes	206 (35.09)	425 (36.02)	0.146	0.703
No	381 (64.91)	755 (63.98)		
Family history of birth defects, n (%)				
Yes	58 (9.88)	61 (5.17)	13.852	< 0.001
No	529 (90.12)	1119 (94.83)		
Active or passive smoking, n (%)				
Yes	345 (58.77)	678 (57.46)	41.379	< 0.001
No	242 (41.23)	502 (42.54)		
Drinking, n (%)				
Yes	18 (3.07)	8 (0.68)	15.425	< 0.001
No	569 (96.93)	1172 (99.32)		
Folic acid supplement, n (%)				
Yes	450 (76.66)	1020 (86.44)	26.812	< 0.001
No	137 (23.34)	160 (13.56)		
Fever, n (%)				
Yes	53 (9.03)	78 (6.61)	3.341	0.068
No	534 (90.97)	1102 (93.39)		
Taking medicine, n (%)				
Yes	129 (21.98)	161 (13.64)	19.837	< 0.001
No	458 (78.02)	1019 (86.36)		
GDM, n (%)				
Yes	16 (2.73)	44 (3.73)	1.202	0.273
No	571 (97.27)	1136 (96.27)		
TORCH infections, n (%)				
Yes	6 (1.02)	2 (0.17)	4.573	0.032 ^b^
No	581 (98.98)	1178 (99.83)		
Industries exposure, n (%)				
Yes	73 (12.44)	42 (3.56)	50.765	< 0.001
No	514 (87.56)	1138 (96.44)		

^a^ Wilcoxon rank test; ^b^ Fisher exact test

### Housing renovation and isolated CHD

As mentioned in Table 2, the case group had a greater probability of being exposed to housing renovations than the control group (25.72% vs. 19.07%). After adjusting for baseline demographic characteristics, exposure to housing renovation was associated with an increased risk for isolated CHD in offspring (adjusted OR = 1.60, 95% CI: 1.23, 2.07). After adjusting for all covariates, the risk of developing isolated CHD was 1.77 (95% CI: 1.34, 2.33).

The association between housing renovation and the substyle of isolated CHD was further analyzed (Table 2). After adjusting for all covariates, housing renovation exposure was associated with an increased risk for VSD and PDA in offspring (VSD: adjusted OR = 1.56, 95% CI: 1.01, 2.41; PDA: adjusted OR = 2.50, 95% CI: 1.41, 4.45). However, there was no significant influence on the other CHD types, such as ASD (adjusted OR = 1.28, 95% CI: 0.67, 2.44), AVSD (adjusted OR = 1.36, 95% CI: 0.65, 2.86), and TOF (adjusted OR = 1.45, 95% CI: 0.65, 3.21).

**Table 2:** Association between housing renovation exposure and isolated CHD

	Indoor renovation ^a^, n (%)			Model 1		Model 2 ^b^		Model 3 ^c^
	Yes	No		Crude OR (95%CI), P value		Adjusted OR (95%CI), P value		Adjusted OR (95%CI), P value
Control	225 (19.07)	955 (80.93)		1.47 (1.16, 1.86), 0.001		1.60 (1.23, 2.07), < 0.001		1.77 (1.34, 2.33), < 0.001
Case	151 (25.72)	436 (74.28)		1.00		1.00		1.00
Main CHD types								
VSD	45 (24.46)	139 (75.54)		1.37 (0.95, 1.98), 0.089		1.41 (0.94, 2.12), 0.095		1.56 (1.01, 2.41), 0.046
ASD	16 (21.92)	57 (78.08)		1.19 (0.67, 2.11), 0.549		1.19 (0.65, 2.17), 0.570		1.28 (0.67, 2.44), 0.405
PDA	24 (32.00)	51 (68.00)		2.00 (1.20, 3.31), 0.007		1.94 (1.14, 3.28), 0.014		2.50 (1.41, 4.45), 0.002
AVSD	12 (24.49)	37 (75.51)		1.38 (0.71, 2.68), 0.348		1.26 (0.63, 2.52), 0.515		1.36 (0.65, 2.86), 0.414
TOF	11 (24.44)	34 (75.56)		1.37 (0.69, 2.75), 0.371		1.35 (0.64, 2.87), 0.428		1.45 (0.65, 3.21), 0.365

^a^ 213 women (18.05%) were exposed to housing renovation at home, and 12 women (1.02%) were exposed to housing renovation at work in the control group; 146 (24.87%) were exposed to housing renovation at home, and 5 (0.85%) were exposed to housing renovation at work in the case group

^b^ Model 2 adjusted maternal age, BMI, education, ethnicity, residence, family wealth index

^c^ Model 3 adjusted maternal age, BMI, education, ethnicity, residence, family wealth index, parity, abortion history, family history of birth defects, active or passive smoke, drinking, folic acid supplement, fever, taking medicine, GDM, TORCH infections and industries exposure

VSD: ventricular septal defect; ASD:atrial septal defect; PDA: patent ductus arteriosus; AVSD: atrioventricular septal defect; TOF: tetralogy of Fallot

### Sensitivity analyses

In addition, the relationship between isolated CHD and exposure to housing renovations was investigated in various demographic subgroups. Housing renovation exposure was associated with a higher risk of isolated CHD in all subgroups, and this association was statistically significant for women aged < 30 years and ≥ 30 years, above high middle school education level, rural or urban residence, medium or high family wealth index, first birth, abortion history or none abortion. Housing renovation exposure was not significantly interacted with any of the covariates for isolated CHD (Table 3).

Additionally, propensity scores for housing renovation exposure were calculated for each participant using multivariable logistic regression, which was used to match 423 (72.06% of 587) cases with 423 controls. After matching, the case group and the control group were similar with regards to all of the baseline covariates except education level (Supplementary Table 1). After propensity scores matching, exposure to housing renovation was associated with an increased risk for isolated CHD in offspring (OR = 1.65, 95% CI: 1.21, 2.26) (Supplementary Table 2).

**Table 3:** Association between housing renovation exposure and isolated CHD in subgroups

	Subgroups	Model 1		Model 2 ^a^		P for interaction
		Crude OR (95%CI), P value		Adjusted OR (95%CI), P value		
Maternal age	< 30 years	1.55 (1.17, 2.06), 0.003		1.66 (1.19, 2.33), 0.003		0.850
	≥ 30 years	1.36 (0.89, 2.07), 0.150		2.04 (1.23, 3.36), 0.005		
Education	High middle school and below	1.11 (0.72, 1.72), 0.639		1.61 (0.95, 2.72), 0.076		0.375
	Above high middle school	1.85 (1.39, 2.46), < 0.001		2.01 (1.46, 2.76), < 0.001		
Residence	Rural	1.83 (1.30, 2.59), < 0.001		2.01 (1.38, 2.92), < 0.001		0.598
	Urban	1.49 (1.04, 2.13), 0.029		1.76 (1.17, 2.64), 0.006		
Family wealth index						
	Low	1.07 (0.72, 1.57), 0.747		1.51 (0.94, 2.44), 0.088		0.074
	Medium	1.86 (1.23, 2.82), 0.003		2.19 (1.39, 3.44), < 0.001		
	High	1.99 (1.25, 3.16), 0.004		2.27 (1.38, 3.75), 0.001		
Parity						
	0	1.91 (1.47, 2.50), < 0.001		1.89 (1.41, 2.54), < 0.001		0.479
	≥ 1	1.36 (0.75, 2.49), 0.314		1.62 (0.81, 3.26), 0.176		
Abortion history						
	Yes	1.88 (1.25, 2.83), 0.002		2.16 (1.40, 3.45), 0.001		0.204
	No	1.30 (0.97, 1.73), 0.077		1.68 (1.20, 2.35), 0.001		

^a^ Model 2 adjusted maternal age, BMI, education, ethnicity, residence, family wealth index, parity, abortion history, family history of birth defects, active or passive smoke, drinking, folic acid supplement, fever, taking medicine, GDM, TORCH infections and industries exposure

## Discussion

Based on a case-control study, we explored the association between periconceptional housing renovation exposure and isolated CHD risk in Xi’an, China. It was found that periconceptional maternal exposure to housing renovation was associated with a greater risk of isolated CHD in offspring. In addition, maternal housing renovation exposure was associated with increased risk for VSD and PDA in subtypes for isolated CHD.

Housing renovation operations are becoming more frequent and an increasing range of renovation materials are being created, thanks to China’s economy and urbanization. Housing decoration materials commonly contain laminated board, plywood, ceramic tile, marbles, oil-based paint, latex or acrylic coating, wallpapers, and carpets, which include various kinds of environmentally hazardous materials that have been reported. For example, formaldehyde and volatile organic compounds (VOCs) (benzene, toluene, xylene, and styrene) can be found in plywood, heavy metals, organic solvents, and VOCs are emitted from dyes, and paints [16, 17]. As a result, many environmentally hazardous materials may be released into the room air after housing renovations, for example, Coombs et al. reported that formaldehyde concentrations were significantly higher in post-renovated than in pre-renovated homes (0.03 vs. 0.01 ppm) [18]. Moreover, low-quality decoration materials may release higher environmentally hazardous materials into the room air [19].

Early pregnancy is a critical period in fetal heart development and the environment at conception, and early pregnancy can impact the developing embryo [20, 21]. Our study found that mothers exposed to housing renovation during the periconceptional period were associated with an increased risk of isolated CHD in offspring. Liu Z et al. conducted a small-size case-control study (346 cases and 408 controls) and found that maternal exposure to housing renovation may have an increased risk of CHD (OR = 1.89, 95%CI: 1.29, 2.77) [1]. Our study had a larger sample size, and the results are in agreement with those of the previous study. Nevertheless, Motoki N et al. discovered that maternal exposure to housing renovation during pregnancy was not substantially associated with an increased risk of CHD (OR = 1.08, 95%CI: 0.72, 1.61). Their research was based on the Japan Environment and Children’s Study, which included 67,503 singleton live births [22]. In an above cohort study, the period of housing renovation exposure was defined as full-term pregnancy, including the first, second, and third trimesters of pregnancy. In our study, the period of housing renovation exposure was defined as from 1 year before pregnancy through the first trimester of pregnancy. Because a crucial period in fetal heart development is the first trimester of pregnancy, the different periods of housing renovation may lead to different effects on CHD in offspring, which may cause different results.

The association between housing renovation exposure and CHD may be attributed to organic pollutants and other volatile contaminants being released from decoration materials. According to a prior study, women whose homes had been decorated in the previous three years had higher urinary benzophenone concentrations than women whose homes had not been decorated [23]. Several studies have revealed that maternal occupational exposure to organic solvents, such as acetone, toluene, xylene, and Stoddard solvent or Chlorinated solvents potentially increased the overall risk of CHD and other system anomalies [8–11]. Also, maternal exposure to organic dyes, lacquers, pigments, and paints during early pregnancy was related to higher cardiac anomalies in the fetus [24, 25]. Some studies showed that exposure to benzene, trichloroethylene, and formaldehyde might increase the prevalence of CHD in offspring [9, 26, 27].

Housing renovation may have different effects on different phenotypes of CHD. By investigating the relationship between housing renovation exposure and the major CHD types, it was found that exposure to housing renovation exposure was associated with an elevated risk for VSD and PDA, which was supported by additional studies. Based on a case-control study, which included all infants born in Finland during 1982–1983 with a VSD, J. Tikkanen and O. P. Heinonen found that maternal exposure to organic solvents during the first trimester may increase the risk of VSD in offspring (OR = 1.8, 95% CI: 1.0, 3.4) [28]. Additionally, a case-control study in the Netherlands found that paternal occupational exposure to phthalates was associated with peri-membranous VSD (adjusted OR = 2.84, 95% CI: 1.37, 5.92) in offspring [29]. While paternal alkylphenolic compound exposure and a higher concentration of maternal blood titanium level increased the risk for PDA in the offspring (OR = 1.95, 95% CI: 1.30, 2.93; OR 2.41, 95% CI: 1.34, 4.36) [30, 31]. Although the association between housing renovation and other phenotypes of CHD (ASD, AVSD, and TOF) was not significant in our study, other research has shown housing renovation exposure or occupational polycyclic aromatic hydrocarbons exposure in pregnancy may be associated with an increased risk for the conotruncal heart defect, specifically TOF [1, 32]. Differences in sample sizes, definitions of exposure and CHD phenotypes, and diagnostic capabilities may contribute to inconsistent outcomes.

This case-control study investigated the association between maternal housing renovation exposure and isolated CHD occurrence. Our study found that maternal exposure to interior housing renovation activity during the periconceptional period may be associated with an increased risk of isolated CHD in offspring. This study added to evidence of the association between housing renovation exposure and the risk of isolated CHD. However, some limitations should be acknowledged. First, although we have established selection criteria for including controls, the selection bias cannot be fully controlled in a hospital-based case-control study. Second, our study did not investigate the specific kinds of housing renovation exposures and the timing of exposures, then we could not perform some sensitivity analysis. Third, even though we account for potential confounders using multivariable regressions and propensity values matching, unmeasured or hidden confounders (such as maternal occupational exposure to solvents/formaldehyde and genetic factors) may still impact the results. Finally, we need to establish a large population cohort with specific ecological contaminants and chemical exposure assessment to confirm the relationship between maternal housing renovation and isolated CHD.

## Conclusions

This study found that maternal exposure to housing renovation during the periconceptional period was associated with an increased risk of isolated CHD in offspring. In addition, living in a newly redecorated house may increase the fetus’ risk of VSD and PDA. Therefore, in order to prevent isolated CHD in infants, it may be beneficial to avoid living in a renovated house from 12 months before pregnancy through the first trimester of pregnancy.

## Electronic supplementary material

Below is the link to the electronic supplementary material.

Supplementary Material 1

## Data Availability

The raw data supporting the conclusions of this article will be made available by the authors, without undue reservation.
