# Supplementary material for: Maternal exposure to housing renovation during the periconceptional period and the risk of offspring with isolated congenital heart disease: a case-control study
Source: Environ Health. 2023 Apr 18;22:37. doi: 10.1186/s12940-023-00990-z (PMC10111801; doi:10.1186/s12940-023-00990-z)
Supplement: Supplementary file 1 — Supplementary Material 1 [file 12940_2023_990_MOESM1_ESM.docx]

**Supplementary table** 1 Baseline characteristics of participants between case group and control group after propensity scores matching

|  | Case group  n=423 | Control group  n=423 | t/χ^2^ /Z value | *P* value |
| --- | --- | --- | --- | --- |
| Maternal age, n (%) | 28.54 ± 5.14 | 28.72 ± 4.35 | -0.907 | 0.364 ^a^ |
| Maternal BMI | 20.52 ± 2.40 | 20.54 ± 2.18 | -0.216 | 0.829 ^a^ |
| Education, n (%) |  |  |  |  |
| Primary school and below | 6 (1.42) | 2 (0.47) | 65.487 | <0.001^b^ |
| Junior middle school | 11 (2.60) | 53 (12.53) |  |  |
| High middle school | 149 (35.22) | 68 (16.08) |  |  |
| Above high middle school | 257 (60.76) | 300 (70.92) |  |  |
| Ethnicity, n (%) |  |  |  |  |
| Han | 417 (98.58) | 417 (98.58) | 0.000 | 1.000 |
| Other | 6 (1.42) | 6 (1.42) |  |  |
| Residence, n (%) |  |  |  |  |
| Rural | 240 (56.74) | 231 (54.61) | 3.888 | 0.533 |
| Urban | 183 (43.26) | 192 (45.39) |  |  |
| Family wealth index, n (%) |  |  |  |  |
| Low | 176 (41.61) | 178 (42.08) | 0.049 | 0.976 |
| Medium | 135 (31.91) | 132 (31.21) |  |  |
| High | 112 (26.48) | 113 (26.71) |  |  |
| Parity, n (%) |  |  |  |  |
| 0 | 299 (70.69) | 307 (72.58) | 0.747 | 0.688 |
| 1 | 114 (26.95) | 109 (25.77) |  |  |
| ≥2 | 10 (2.36) | 7 (1.65) |  |  |
| Abortion history, n (%) |  |  |  |  |
| Yes | 158 (37.35) | 162 (38.30) | 0.080 | 0.777 |
| No | 265 (62.65) | 261 (61.70) |  |  |
| Family history of birth defects, n (%) |  |  |  |  |
| Yes | 28 (6.62) | 36 (8.51) | 1.082 | 0.298 |
| No | 395 (93.38) | 387 (91.49) |  |  |
| Active or passive smoking, n (%) |  |  |  |  |
| Yes | 223 (52.72) | 223 (52.72) | 0.000 | 1.000 |
| No | 200 (47.28) | 200 (47.28) |  |  |
| Drinking, n (%) |  |  |  |  |
| Yes | 7 (1.65) | 5 (1.18) | 0.338 | 0.561 |
| No | 416 (98.35) | 418 (98.82) |  |  |
| Folic acid supplement, n (%) |  |  |  |  |
| Yes | 351 (82.98) | 347 (82.03) | 0.131 | 0.717 |
| No | 72 (17.02) | 76 (17.97) |  |  |
| Fever, n (%) |  |  |  |  |
| Yes | 28 (6.62) | 28 (6.62) | 0.000 | 1.000 |
| No | 395 (93.38) | 395 (93.38) |  |  |
| Taking medicine, n (%) |  |  |  |  |
| Yes | 85 (20.09) | 88 (20.80) | 0.065 | 0.798 |
| No | 338 (79.91) | 335 (79.20) |  |  |
| GDM, n (%) |  |  |  |  |
| Yes | 11 (2.60) | 13 (3.07) | 0.172 | 0.679 |
| No | 412 (97.40) | 410 (96.93) |  |  |
| TORCH infections, n (%) |  |  |  |  |
| Yes | 1 (0.24) | 2 (0.47) |  | 1.000 ^b^ |
| No | 422 (99.76) | 421 (99.53) |  |  |
| Industries exposure, n (%) |  |  |  |  |
| Yes | 33 (7.80) | 29 (6.86) | 0.278 | 0.598 |
| No | 390 (92.20) | 394 (93.14) |  |  |

^a^ Wilcoxon rank test; ^b^ Fisher exact test.

**Supplementary table** 2 Association between housing renovation exposure and isolated CHD after propensity scores matching

|  | Indoor renovation, n (%) | |  | OR (95%CI), P value |
| --- | --- | --- | --- | --- |
|  | Yes | No |  |  |
| Controls | 84 (19.86) | 339 (80.14) |  | 1.00 |
| Case | 123 (29.08) | 300 (70.92) |  | 1.65 (1.21, 2.26), 0.002 |
